# AI-enabled cybersecurity framework for future 5G wireless infrastructures

**DOI:** 10.1038/s41598-026-37444-8

**Published:** 2026-02-03

**Authors:** Asad Alam, Asif Umer, Insaf Ullah, Ahmed Alsayat

**Affiliations:** 1 Faculty of Technology, University of Portsmouth, Portsmouth, UK; 2 Department of Computer Science & IT, Hazara University, Mansehra, KPK Pakistan; 3 Institute for Analytics and Data Science, University of Essex, Colchester, CO4 3SQ UK; 4 Department of Computer Science, College of Computer and Information Sciences, Jouf University, 72341 Sakaka, Saudi Arabia

**Keywords:** 5G security, AI, Federated learning, Zero trust, Network slicing, Intrusion detection, Energy science and technology, Mathematics and computing

## Abstract

The deployment of fifth-generation (5G) wireless networks is transforming digital connectivity through ultra-low latency, high data rates, and massive device support. However, enabling technologies such as network slicing, virtualization, edge computing, and dense Internet of Things (IoT) integration significantly expand the attack surface, necessitating advanced cybersecurity strategies. This study proposes a comprehensive multi-layered cybersecurity framework tailored for 5G infrastructures. The framework incorporates device-level trust validation, secure network slice configuration and isolation, dynamic policy enforcement at the orchestration layer, and AI-driven threat detection to provide end-to-end protection across the 5G architecture. Unlike traditional reactive security models, the proposed approach adopts security-by-design principles to proactively mitigate threats. The framework’s effectiveness is evaluated through extensive simulations and benchmarking against established standards, including the NIST Zero Trust Architecture and 3GPP TS 33.501. Results demonstrate a threat detection rate of up to 97.6%, low-latency performance under high-load and adversarial conditions, and scalable operation with large-scale device connectivity. Despite these results, challenges remain in ensuring consistent policy enforcement across distributed edge nodes, achieving interoperability among heterogeneous devices, and balancing performance with stringent security requirements. The study concludes by highlighting future research directions, including quantum-resilient cryptography and self-healing, AI-enhanced security mechanisms, to address evolving threats in future 6G networks.

## Introduction

**Fig. 1:**
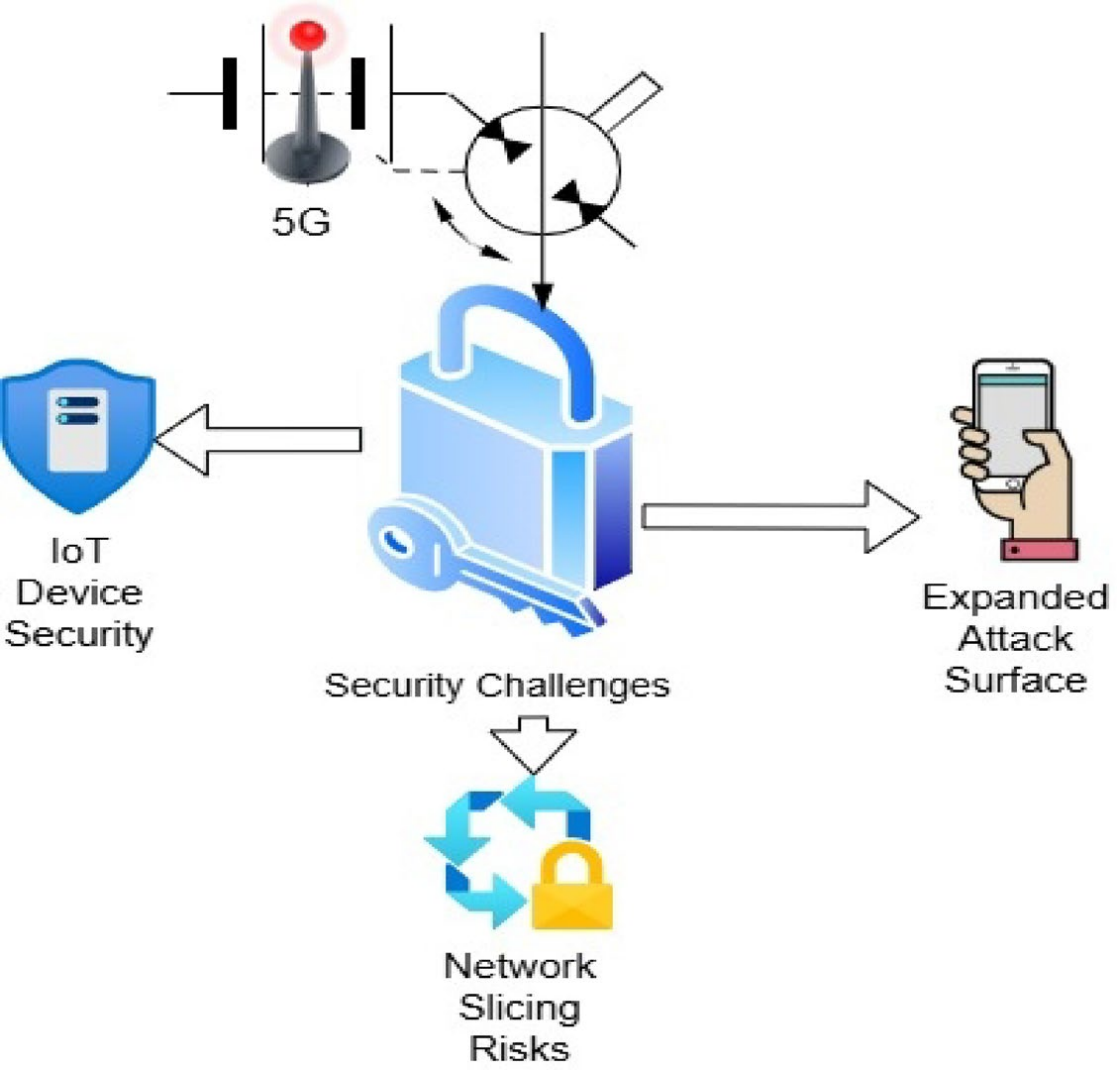
A Comprehensive Overview of Security Challenges in 5G Networks. This figure illustrates the multi-layered security challenges introduced by 5G network architecture, highlighting vulnerabilities across the device, access, edge, and core layers. Key issues include insecure IoT device integration, dynamic attack surfaces due to network slicing and virtualization, latency-sensitive threats at the edge, and the complexity of enforcing uniform security policies in a decentralized environment. The figure also emphasizes emerging risks associated with dense device connectivity, heterogeneous technologies, and the need for real-time threat detection and mitigation mechanisms. These challenges underscore the necessity for proactive, end-to-end, and adaptive cybersecurity frameworks tailored for 5G systems.

Industry 5G, also known as the fifth generation of wireless technology, represents a significant advancement in mobile communications. Unlike earlier generations from 1 to 4G, which primarily focused on enhancing internet speed, voice quality, and basic mobile connectivity, 5G introduces a fundamentally new type of network designed to support a highly connected digital world^1^. 1G introduced analog voice communication in the 1980s; 2G brought digital voice and SMS; 3G enabled mobile internet access; and 4G revolutionized mobile broadband with high-speed data and video streaming. It offers incredibly fast speeds, reaching up to 10 gigabits per second, and extremely low latency, sometimes as little as one millisecond. This level of real-time responsiveness is crucial for exciting new technologies such as smart cities, self-driving cars, advanced industrial systems, remote surgeries, and extensive networks of smart devices. One standout feature of 5G is called network slicing, which allows multiple virtual networks to operate on a single shared physical network^2^. Each slice can be tailored to meet the needs of a specific application. For example, one slice may focus on streaming high-quality video, while another can be designed for applications like autonomous vehicles that require very quick response times. To improve performance even further, 5G utilizes advanced tools like Massive MIMO, which stands for multiple input and multiple output, and beamforming, which helps send stronger and more focused signals^3^. It also employs very high frequency bands, known as millimeter waves, that are above 24 gigahertz. These frequencies enable much faster data transmission. Another important feature is edge computing, which involves processing data closer to its source instead of sending everything to the cloud. This helps reduce delays, which is especially critical in situations where time is of the essence, such as in emergency services or industrial safety systems. 5G is designed to support a wide variety of uses, including enhanced mobile broadband for faster internet, ultra-reliable communication with very low latencies, and the ability to connect a vast number of machines and devices simultaneously^4^. This makes it beneficial across various industries and everyday scenarios. At the same time, the new design of 5G introduces fresh security and privacy concerns. Because the network is more open, flexible, and distributed, it becomes increasingly vulnerable to attacks^5^. The old security models no longer suffice, as 5G networks are now programmable, virtual, and intricately connected to countless devices and external services^6^. This complex structure, involving everything from smart sensors to mobile apps and cloud services, creates numerous weak points that attackers could attempt to exploit. Moreover, 5G security has a global aspect, as countries have concerns about threats such as cyberattacks from rival nations, espionage, and ransomware targeting critical systems. Figure 1 illustrates the security challenges in the 5G architecture.

Open-RAN (Open Radio Access Network) is an emerging approach in mobile network architecture that promotes interoperability and vendor neutrality by disaggregating hardware and software components. While it is gaining traction in specific 5G deployments, particularly in regions prioritizing flexible and cost-efficient infrastructure, it is not yet universally adopted across global networks. Many operators continue to rely on traditional, vertically integrated RAN solutions due to concerns over maturity, security, and performance consistency in Open-RAN implementations. The primary objective of this study is to analyze the unique security challenges introduced by the architecture of 5G networks, which differ significantly from previous generations due to their decentralized, software-based, and highly connected structure. It also aims to examine existing cybersecurity frameworks, protocols, and technologies to evaluate how well they address the specific needs of 5G systems. Furthermore, the study seeks to explore the potential of emerging solutions, such as artificial intelligence-based threat detection, blockchain integration, and zero trust security models, assessing their relevance and effectiveness in protecting 5G infrastructure. Finally, the research intends to propose a conceptual model or a set of best practices for designing cybersecurity strategies specifically suited to the complex and evolving nature of 5G environments. This research investigates the laws and technologies related to 5G cybersecurity, focusing on how 5G networks are constructed, including software-based networking and virtual network functions. The study also reviews regulations and privacy laws that companies must follow. It highlights the importance of robust 5G security across various sectors, particularly in smart healthcare systems. Furthermore, it emphasizes the necessity for protection in systems that connect logistics, supply chains, and factories in modern industries like Industry 4.0. This work focuses on a multi-layered 5G security architecture integrating device attestation, trust scoring, slice-aware isolation, zero-trust orchestration, and AI-driven threat detection. Other areas such as physical security and general SDN/NFV threats are referenced but not elaborated in detail, to maintain a clear focus on architectural integration and AI-enabled protection mechanisms.

### Contributions

This study makes the following key contributions to the field of 5G network security:

- *Proposes a novel multi-layered cybersecurity framework* for 5G networks that integrates trust scoring, edge-based federated threat detection, and dynamic policy enforcement to ensure robust and scalable protection.
- *Introduces a customized trust evaluation model* that leverages federated learning to enhance both the scalability and privacy of threat detection, minimizing data exposure while enabling distributed intelligence.
- *Implements and evaluates the proposed framework* using Mininet-WiFi for network emulation and a hybrid CNN-LSTM deep learning model for real-time threat detection.
- *Demonstrates superior performance*, achieving a threat detection accuracy of 97.6%, maintaining latency below 6.5 ms even during active attack scenarios, and supporting over one million simultaneously connected devices.

The rest of this paper is organized as follows. Section “Literature review” presents a comprehensive review of related work, discussing existing methods, frameworks, and challenges in the domain of 5G cybersecurity. Section “Proposed framework” introduces the proposed multi-layered cybersecurity framework, detailing its architecture, core components, and operational mechanisms. Section “Evaluation and results” outlines the experimental setup and presents the evaluation results, demonstrating the performance, accuracy, and scalability of the proposed approach. Finally, Section “Conclusion” concludes the paper by summarizing the key findings and proposing future research directions, including the integration of advanced technologies to address anticipated security challenges in 6G networks.

## Literature review

The transition from 4G LTE to 5G represents a significant advancement in telecommunications, delivering ultra-high speeds, reduced latency, and enhanced bandwidth capacity. Research such as^7^ highlights 5G’s critical role in improving Quality of Service (QoS) and enabling emerging technologies, including the Internet of Things (IoT), Virtual Reality (VR), and autonomous systems. This study also elaborates on the 5G architecture and its associated security implications, emphasizing the need for future investigations into energy efficiency, IoT integration, and advanced communication protocols to support evolving digital ecosystems. While^7^ discusses security challenges in 5G, it does not incorporate AI-driven anomaly detection for edge computing, which our framework addresses. In^8^, the authors examine the structural evolution of the 5G core network and propose security considerations pertinent to new technologies such as Network Function Virtualization (NFV), Software Defined Networking (SDN), and Multi-Access Edge Computing (MEC). The increased architectural complexity introduces novel attack vectors that render traditional security mechanisms inadequate. Their analysis identifies vulnerabilities across multiple networks protocols and outlines key security challenges, emphasizing the necessity for enhanced visibility and control within 5G environments. The study further advocates for increased transparency and collaborative research, particularly considering the proprietary nature of many carrier infrastructures. The study in^8^ focuses on NFV/SDN security but lacks real-time predictive threat detection; our model integrates AI at the edge for proactive responses.

In^9^, the author proposed a comprehensive categorization of cybersecurity threats within 5G networks, including physical, local, and remote attack vectors. While 5G capabilities such as enhanced Mobile Broadband (eMBB), massive Machine-Type Communications (mMTC), and Ultra-Reliable Low Latency Communications (URLLC) improve performance metrics, they simultaneously introduce vulnerabilities that compromise the core principles of Confidentiality, Integrity, and Availability (CIA). The study highlights those applications dependent on Vehicle-to-Vehicle (V2V) communication, Augmented Reality (AR), and IoT-based services significantly expand the threat landscape, primarily due to the absence of standardized protocols and the proliferation of interconnected devices. In^9^ categorizes 5G cyber threats comprehensively but does not propose a unified multi-layered mitigation framework; our approach combines device, RAN, edge, and core layers for holistic protection. The work in^10^ analyzes the multifaceted impact of 5G on network architecture, business models, and cybersecurity. The authors argue that the software-centric nature of 5G networks introduces new classes of vulnerabilities, especially as frequent software updates become the norm. They stress the imperative for cybersecurity strategies to evolve alongside technological advancements, given 5G’s increasing integration into critical infrastructure. Furthermore, the study notes the underutilization of simulation tools and testbeds that are essential for pre-deployment identification and mitigation of security flaws. Although^10^ highlights software vulnerabilities in 5G networks, it does not exploit machine learning for anomaly detection; our framework leverages AI to identify threats dynamically.

In^11^, approaches 5G cybersecurity from a strategic and systematic perspective, identifying critical threats such as exploitation of network slicing, vulnerabilities in edge computing, and supply chain risks unique to 5G ecosystems. The authors propose a multi-layered cybersecurity framework that integrates traditional defenses, including encryption and authentication, with emerging approaches such as AI-driven threat detection. They underscore the importance of proactive security design to effectively address the dynamic and evolving nature of cyber threats, warning that failure to anticipate these challenges may compromise network reliability. The multi-layered strategy in^11^ integrates traditional defenses with AI detection but does not scale efficiently to massive device deployments; our framework is tested for ≥ 1 million devices/km^2^, supporting ultra-dense IoT networks.

### Cybersecurity threats in 5G networks

5G technology brings faster internet and connects many more devices than before. But with these improvements come new security risks. The way 5G networks are built using lots of software and new technologies like network slicing and edge computing makes them more open to attacks than older networks. There are many ways hackers can try to break in, especially because 5G connects so many devices like sensors and smart gadgets. Also, since 5G will support important services like healthcare and transportation, keeping it safe is very important. Knowing the kinds of threats 5G faces helps us build better protections to keep networks and users secure.

#### 5G threat landscape

The 5G network connects various devices and systems, creating a vast and complex threat landscape. This includes attacks on physical devices, software vulnerabilities, and risks from remote hackers. Because 5G supports new technologies such as smart cities, autonomous vehicles, and the internet of things, attackers have more avenues to inflict harm. The diversity of devices and applications means security weaknesses can arise from many sources, including connected sensors, mobile apps, and cloud services. Understanding this wide range of threats is crucial for protecting the entire 5G network and ensuring user safety. The introduction of 5G wireless networks has significantly transformed connection speeds and security management. The decentralized architecture, virtualization of functions, extensive edge computing, and software-defined networking set 5G apart from its predecessors. While these innovations enhance network performance and flexibility, they also increase the attack surface, heightening possible security risks. Given that 5G underpins critical sectors such as healthcare, energy, finance, and transportation, ensuring robust cybersecurity is vital. Due to its open and dynamic nature, 5G networks are more vulnerable to cyber threats. These threats range from distributed denial of service attacks to state-sponsored intrusions aimed at data theft or infrastructure damage^12^. While^12^ focuses on distributed denial-of-service attacks, it does not consider edge-based federated learning for real-time mitigation, which our framework incorporates.

#### Physical layer vulnerabilities

5G is a highly advanced digital communication system, it remains dependent on physical infrastructure components, including cell towers, base stations, antennas, and fiber-optic links. These assets are frequently deployed in open or minimally supervised environments, making them vulnerable to physical attacks and unauthorized access. Malicious actors may attempt to damage network equipment, introduce compromised firmware or malware, exfiltrate sensitive data, or deliberately disrupt service availability. Millimeter-wave transmissions, which enable high-capacity data transfer, are susceptible to obstruction and targeted interference. Furthermore, the widespread deployment of small cells to enhance coverage density significantly increases the number of physical assets requiring protection, thereby expanding the potential attack surface. Inadequate physical security controls may allow these components to be exploited for surveillance, service disruption, or denial-of-service attacks. Consequently, robust physical security mechanisms, access control policies, and continuous monitoring are essential to ensuring the resilience and reliability of 5G network infrastructure^13^. Physical layer vulnerabilities are identified in^13^, yet there is no mechanism for automated local response; our model enables self-healing actions at the edge for immediate fault containment.

#### Risks in radio access networks (RAN) and edge computing

The intermediate Radio Access Network (RAN) connects user devices to the main network. In 5G, a newer type called Open RAN (O-RAN) is used. It is based on software and supports equipment from different manufacturers. This open approach helps improve innovation and efficiency but also increases security risks. The software, APIs, and network interfaces can have weaknesses that hackers might use to steal data or install malware. 5G also uses edge computing to process data closer to where it is created, which helps reduce delay^14^. But because edge devices are spread out in many places, they are harder to protect. Each edge node acts like a small data center that could be hacked, set up incorrectly, or have security flaws in its software. Since these nodes are often placed in third-party locations like smart factories and hospitals and are not closely monitored, attackers can use them to target important systems with little resistance. Open-RAN security issues discussed in^14^ are largely theoretical; our framework provides practical AI-driven detection and response across multi-vendor O-RAN environments.

#### Vulnerabilities in SDN/NFV and network slicing

5G networks leverage Network Function Virtualization (NFV) and Software-Defined Networking (SDN) to dynamically control and orchestrate network resources. These advancements enhance efficiency and scalability but also shift numerous network management tasks to software layers, increasing their susceptibility to configuration errors and vulnerabilities. By decoupling the control plane from the data plane, SDN centralizes network control in the controller. If an attacker gains access to the controller, they can manipulate data flows, conduct surveillance, or launch denial-of-service attacks^15^. Network slicing enables multiple virtual networks to operate simultaneously on the same physical infrastructure in 5 G. However, flaws in the slice isolation mechanisms can allow threats to propagate laterally across slices, despite their logical separation. For example, compromising an IoT device-specific slice might give an attacker access to more sensitive slices handling mission-critical applications such as industrial automation or remote surgery. Security policy enforcement becomes increasingly complex when slices span multiple vendors and infrastructure layers, creating new attack surfaces. The inclusion of third-party virtual network functions (VNFs) further exacerbates these risks. VNFs are susceptible to supply chain attacks if their integrity is not thoroughly verified. A compromise of even a single VNF can enable persistent attacks that evade detection. SDN/NFV vulnerabilities are analyzed in^15^, but the study does not provide adaptive, AI-powered orchestration; our approach dynamically enforces policies based on real-time threat intelligence.

#### Threats from IoT devices and endpoints

5G is expected to support up to one million devices in one square kilometer, which will enable massive machine-type communication. This very high number of devices creates serious cybersecurity challenges. Many 5G Internet of Things devices are low-cost and have limited resources. They often use weak or default passwords, making them easy targets for attackers. These devices can be controlled by botnets to launch attacks that overwhelm networks, steal data, or move through the network without permission. It is hard to set security standards because the devices are very different, such as sensors, drones, smart meters, and wearables^16^. Many of these devices are no longer supported by their makers or cannot be updated, so they remain open to new types of attacks. For example, an insecure wearable device could allow someone to change health information or access private patient data without permission. Also, because many devices work in places where they can be physically stolen or tampered with, attackers might find sensitive information or encryption keys. To stop unauthorized access, it is important to have strong methods for device authentication and identity management.

#### State-sponsored attacks, ransomware, and supply chain threats

Many governments see 5G networks as important for national power and control because of their role in global politics. As a result, 5G networks are becoming the target of more complex and frequent cyberattacks supported by governments. These attacks often do not cause immediate damage but aim to gather information over time, spy on users, or secretly set up ways to cause future harm. Attackers often use hidden and advanced methods, known as Advanced Persistent Threats (APTs), to take control of important parts of the network. Ransomware, which was once mostly used against regular IT systems, is now also targeting the telecom sector. A well-planned ransomware attack can shut down tools that manage the network or take control of data centers^17^. Malware can also be secretly added during the production or setup of the network. Later, it can be activated to steal data, stop services, or change important settings. Other risks include fake firmware updates, untested software from outside companies, and weak checking of vendor software. These risks must be controlled through strong security practices.

#### Attack vectors: jamming, spoofing, and MITM

Because 5G relies on wireless communication, it is open to radio frequency (RF) attacks such as jamming, spoofing, and man-in-the-middle (MITM) attacks. Jamming is still a powerful method that blocks communication by flooding the signal with noise^18^. Since 5G uses millimeter wave (mmWave) frequencies, which are more easily disrupted, jamming can cause serious problems. Attackers may focus on important parts of the network, such as the radio access network (RAN) or edge connections, to slow down service or force users onto weaker backup systems. In spoofing attacks, a bad actor pretends to be a trusted device or base station to trick users into sharing private information. For example, fake base stations, also known as “stingrays”, can infect mobile phones with spyware or capture calls and messages. These attacks are especially dangerous for secure financial services or critical systems that depend on 5 G. Man-in-the-middle attacks use weak encryption or poor session management to secretly listen to or change messages between two real users. Because 5G has a complex and constantly changing structure, it brings new types of security risks. Protecting 5G from physical threats, radio-level attacks, and advanced cyber threats in software-defined networking, edge computing, and virtual systems requires a strong and forward-looking plan. Old-style security that protects only the network’s edges is not enough. Every layer of the network should use constant monitoring, zero-trust security models, and security built into the design from the start.

#### Emerging technologies in 5G security

As 5G networks improve digital systems, old security methods are no longer enough. To protect these advanced and flexible networks, security solutions need to be intelligent and able to adapt. Because cyber threats are growing, new methods like blockchain, zero trust security, and artificial intelligence are now being used to meet 5G security needs. These tools help find, prevent, and respond to attacks in real time. They are designed to handle large scale, speed, and variety of devices in 5G networks. Artificial intelligence and machine learning are important for finding threats in 5G networks. These systems can study huge amounts of data quickly and notice strange behavior that older tools may miss. In 5G, artificial intelligence can be used in many parts of the network to watch how people and devices act, track traffic patterns, and find problems in communication^19^. These systems learn what normal behavior looks like and raise alerts when something changes. For example, if a smart device starts sending too much data or connects to unknown places, the system can block it. Artificial intelligence can also detect attacks from botnets by comparing the behavior of devices with known threat patterns. Learning models can be trained to recognize past attack steps and help stop future ones before they start. These systems use information from network traffic and apply deep learning to find early signs of threats like denial-of-service attacks. A smart threat detection system usually collects network data, picks out important details, and uses learning models to decide if there is a problem. This improves the time it takes to detect and fix issues. Zero trust security is another new method that does not automatically trust anyone or any device. Instead, it checks who they are, where they are, and if they are safe before allowing access to the network. This is different from old systems that trusted users after they logged in once. Zero trust is useful for 5G because it has many kinds of connected devices. It divides the network into smaller parts and keeps checking identity and safety even after access is granted. For example, a remote device trying to open a health app may be blocked or limited based on where it is or how it behaves. Identity and access systems help make these decisions, and zero trust works well with 5G’s modern technologies. Blockchain can also help improve 5G security by making records safe and verifying identity^20^. It keeps a shared digital record of events that cannot be changed. This removes the need for a single trusted authority. In networks with many devices, blockchain helps manage identity and store records of user actions and device connections. Some systems allow smart devices to create their own identity without needing central control. Blockchain can also record events like device login or network use, which helps during audits and investigations. Some types of blockchain use smart contracts to manage services and can automatically act if a service is not working correctly. Blockchain is also helpful for managing shared radio resources and agreements between service providers. It can be used in many parts of the network to keep access and data safe.

Edge computing, a critical enabler of ultra-low latency and localized processing in 5G networks, is fundamentally transforming network architectures. However, the decentralized placement of computing resources, often in semi-trusted or physically exposed environments, introduces new security challenges. Edge nodes, which support real-time data analytics, machine learning tasks, and content catching near-users, have become high-value targets for cyberattacks.

The primary objective of 5G edge security is to ensure the confidentiality, integrity, and availability of both data-in-transit and data-at-rest at the edge, while maintaining seamless service delivery^21^. Trusted Execution Environments (TEEs) are deployed at the User Equipment (UE) and MEC nodes to secure critical computations. Secure boot mechanisms are applied at device startups to ensure firmware integrity, while lightweight encryption protects inter-container communication in edge computing environments. These mechanisms are triggered during device onboarding, network slice initialization, and inter-service data exchange. Furthermore, the prevalent use of containers and microservices in edge environments increases the attack surface, raising the risk of code injection, container breakout, or runtime compromise by adversaries. Consequently, robust and adaptive security mechanisms are essential to safeguard edge infrastructure in 5G ecosystems. To reduce these risks, systems must detect weak points and stop dangerous behavior while the system is running.

**Fig. 2:**
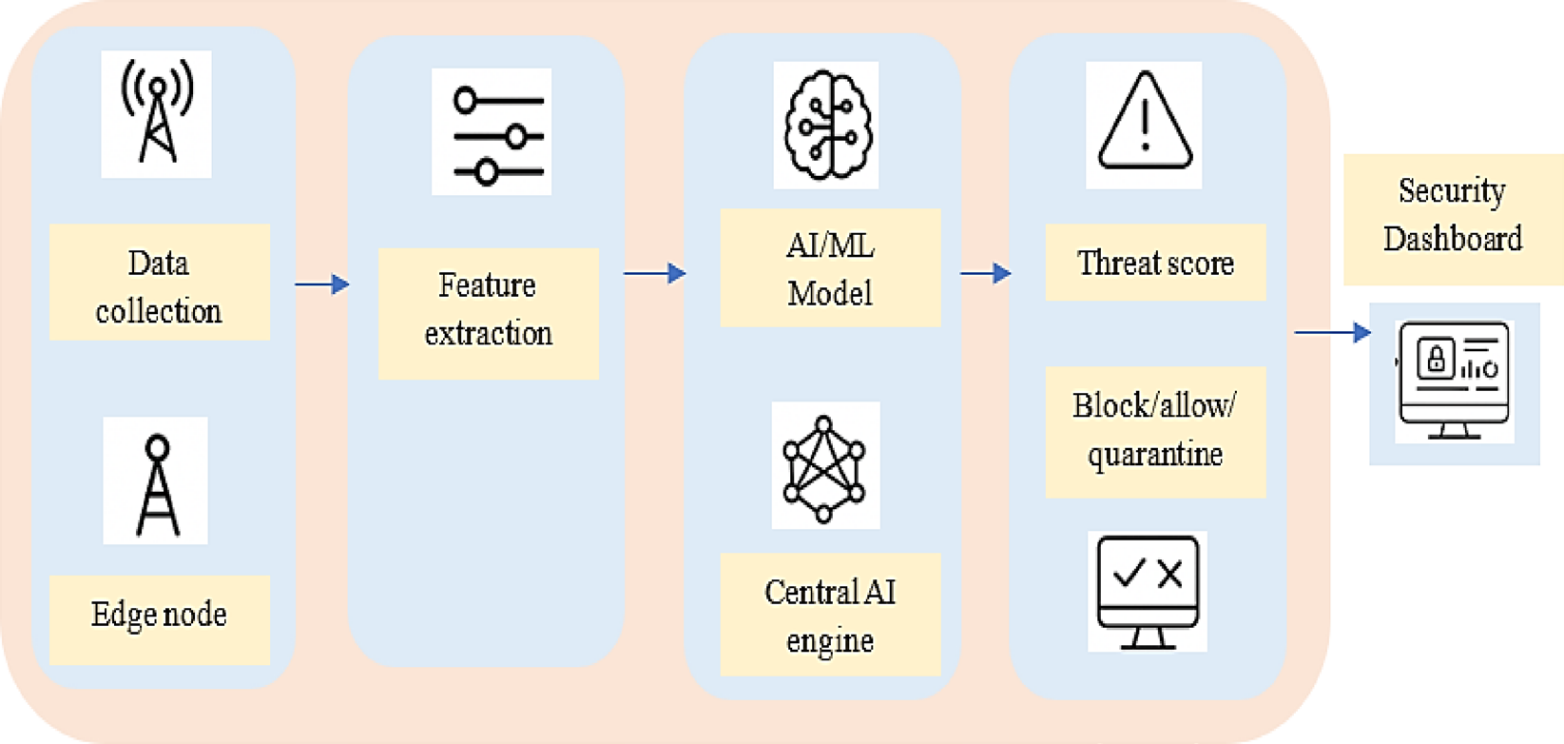
Content Flow for AI-Driven Intrusion Detection in Edge Networks. The diagram represents how AI algorithms process, detect, and respond to intrusion attempts at the edge. Edge devices analyze real-time data streams, apply security rules using trained models, and forward aggregated insights to the central server. This decentralized detection mechanism enables rapid response while maintaining data privacy and reducing core network load.

Artificial intelligence (AI) significantly enhances edge security by enabling the implementation of intelligent, context-aware rules that can detect and respond to threats locally. As illustrated in Fig. 2, security mechanisms deployed at the edge can identify and mitigate malicious activity before it propagates to the core network. For instance, if a connected vehicle transmits anomalous or suspicious data, the edge device can immediately block communication from that vehicle and issue an alert to the central security system. This localized response helps contain threats early and prevents broader network compromise. A modern and privacy-preserving technique known as federated learning further strengthens edge security. Federated learning enables edge devices to collaboratively train machine learning models without sharing raw or sensitive data with a central server. This approach is particularly beneficial in domains involving private healthcare or financial information, where data confidentiality is critical. In an efficiently orchestrated framework, edge devices first analyze local data and apply AI-driven rules to detect anomalies or enforce policies. They then transmit only the aggregated model updates or insights, not the original data, to the central system, which uses this input to make informed, large-scale security decisions^22^. Despite these advantages, several challenges hinder the full realization of AI-based edge security. Resource constraints on edge devices, such as limited computational power, memory, and energy, can affect the performance of complex AI models. Heterogeneity among devices and platforms complicates the deployment and interoperability of federated learning systems. Additionally, ensuring the integrity and trustworthiness of model updates in federated learning is a non-trivial task, as malicious participants may attempt to poison the model. Latency and synchronization issues between distributed nodes can also impact the accuracy and timeliness of threat detection. Addressing these challenges is critical for building robust, scalable, and secure edge-based AI systems^23–26^.

As shown in Table 1, the proposed AI-driven multi-layered framework significantly outperforms both NIST Zero Trust and 3GPP TS 33.501 across multiple dimensions, including detection accuracy, latency, scalability, and coverage of emerging threats. Existing models provide foundational security guidelines but lack integrated AI-based detection mechanisms, edge intelligence, and real-time adaptive capabilities essential for large-scale 5G and beyond networks.

**Table 1:** Comparative analysis of 5G security frameworks and approaches.

Criteria	NIST zero trust (SP 800–207)	3GPP TS 33.501	AI-based multi-layered proposed framework
Security Focus	Identity verification, least privilege, continuous authentication	Network access security (NAS), service layer, application security	Holistic: AI-driven anomaly detection, secure slicing, orchestration, edge AI
Architecture Scope	Perimeter-less, user/device-centric	5G system architecture-specific	Multi-layer (device, RAN, edge, core, orchestration)
AI/ML Integration	Not natively included	Not included	Deep learning for real-time intrusion detection, anomaly prediction
Real-time Threat Response	Moderate—policy-based decisions	Basic—based on predefined protocol behavior	High—predictive, adaptive, self-healing capabilities
Edge Computing Security	Limited focus	Partial (service-level)	Integrated federated learning and AI detection at the edge
Support for Emerging Technologies	Indirect (works with modern cloud stacks)	SDN/NFV supported; limited AI	Designed for IoT, URLLC, O-RAN, MEC, blockchain, AI/FL
Attack Coverage	Internal threats, lateral movement	Protocol-level threats (e.g., rogue base stations)	Broad: DDoS, spoofing, jamming, VNF compromise, botnets
Device Scalability	Medium—depends on policy engine performance	High—supports mMTC and URLLC	Very high—tested for ≥ 1 million devices/km^2^
Latency Under Attack	Not explicitly defined	Varies with deployment	6.5 ms (under active DDoS)
Threat Detection Accuracy	Not applicable	Not AI-driven	97.6% detection accuracy
Compliance & Standards	US Gov/Federal compliance (SP 800-series)	Telecom-grade global compliance	Compliant with both NIST & 3GPP; 6G-ready extensions proposed
Vulnerability Coverage	Insider threats, authentication	Protocol misuse, signaling storms	Full stack: physical, RAN, SDN/NFV, edge, IoT, APTs

#### Practical deployment challenges and legacy system compatibility

While the proposed multi-layered cybersecurity framework offers robust protection for next generation 5G networks, its deployment in real-world telecom infrastructures presents several practical challenges. One major consideration is the heterogeneity of network environments, where 4G, 3G, and even legacy systems co-exist with modern 5G components. To ensure backward compatibility, our framework adopts a hybrid orchestration strategy that integrates SDN/NFV controllers capable of interfacing with both legacy and next-gen systems through API gateways and protocol translation layers. Furthermore, resource constraints at the edge device levels such as limited processing power and battery life, necessitate lightweight AI models and model compression techniques, such as quantization and knowledge distillation. From a regulatory standpoint, data sovereignty laws and cross-border data flow restrictions remain significant hurdles. To address this, the framework incorporates federated learning and decentralized policy enforcement to ensure that sensitive data remains localized while still benefiting from global threat intelligence. These considerations make the framework not only technically feasible but also adaptable to the complex regulatory and infrastructural realities of real-world telecom environments.

Recent advances in AI-driven 5G security have focused on leveraging machine learning and deep learning techniques to enhance threat detection and response. For example, reinforcement learning has been applied to automate threat hunting and adapt defenses dynamically across virtualized 5G environments, improving resilience against complex, evolving attacks^27^. Interpretable anomaly detection frameworks have been proposed for AI-integrated Open RAN systems, combining recurrent models with explainable AI methods to detect DDoS and other sophisticated threats^28^. Self-adaptive jamming detection mechanisms in AI/ML-enabled O-RAN environments demonstrate continuous model retraining and closed-loop feedback to maintain detection performance under changing signal conditions^29^. Additionally, enhanced real-time intrusion detection using self-attention autoencoders shows promise for RF-level anomaly identification^30^, and AI-based anomaly detection for IoT in smart cities illustrates how federated learning can preserve privacy while securing massive edge networks^31^.

While these AI-driven approaches significantly enhance threat detection and resilience in 5G networks, they also face challenges such as high computational overhead at the edge, dependency on large volumes of labeled data for model training, potential adversarial attacks on AI models, and difficulties in ensuring real-time detection under highly dynamic network conditions^27–31^. Addressing these limitations is crucial for practical deployment in large-scale 5G and Open RAN environments. Unlike existing 3GPP SA3 frameworks, conventional Zero-Trust orchestration models, or ML-based IDS such as Kitsune and DeepIDS, the proposed framework integrates: (i) dynamic trust scoring at the device level, (ii) slice-aware isolation, and (iii) federated learning-driven AI for real-time threat detection. Table 1 presents a direct conceptual comparison with prior works, highlighting the unique integration of these components and their coordinated operation in a 5G environment.

## Proposed framework

As 5G networks become the digital backbone of future societies, ensuring their security is critical. The sheer scale, decentralized nature, and high level of programmability in 5G systems introduce a range of vulnerabilities that traditional security approaches struggle to address^26^. To tackle these challenges, we propose a comprehensive, multi-layered cybersecurity framework for 5G networks. Building on the AVSD-MDLN malware detection model from previous research^23^, this framework incorporates AI-powered analytics, adaptive policy enforcement, real-time threat intelligence, and layered defense strategies to enhance protection across all levels of the network.

The proposed architecture comprises four critical components: Device Security, Network Slicing Protection, Secure Orchestration, and Threat Intelligence and Analytics. Each layer is specifically designed to protect the operational functions within its respective domain. Adhering to the defense-in-depth principle, this multi-layered security model reinforces the overall robustness of the 5G infrastructure by distributing protective mechanisms across all architectural levels, thereby minimizing potential vulnerabilities.

**Fig. 3:**
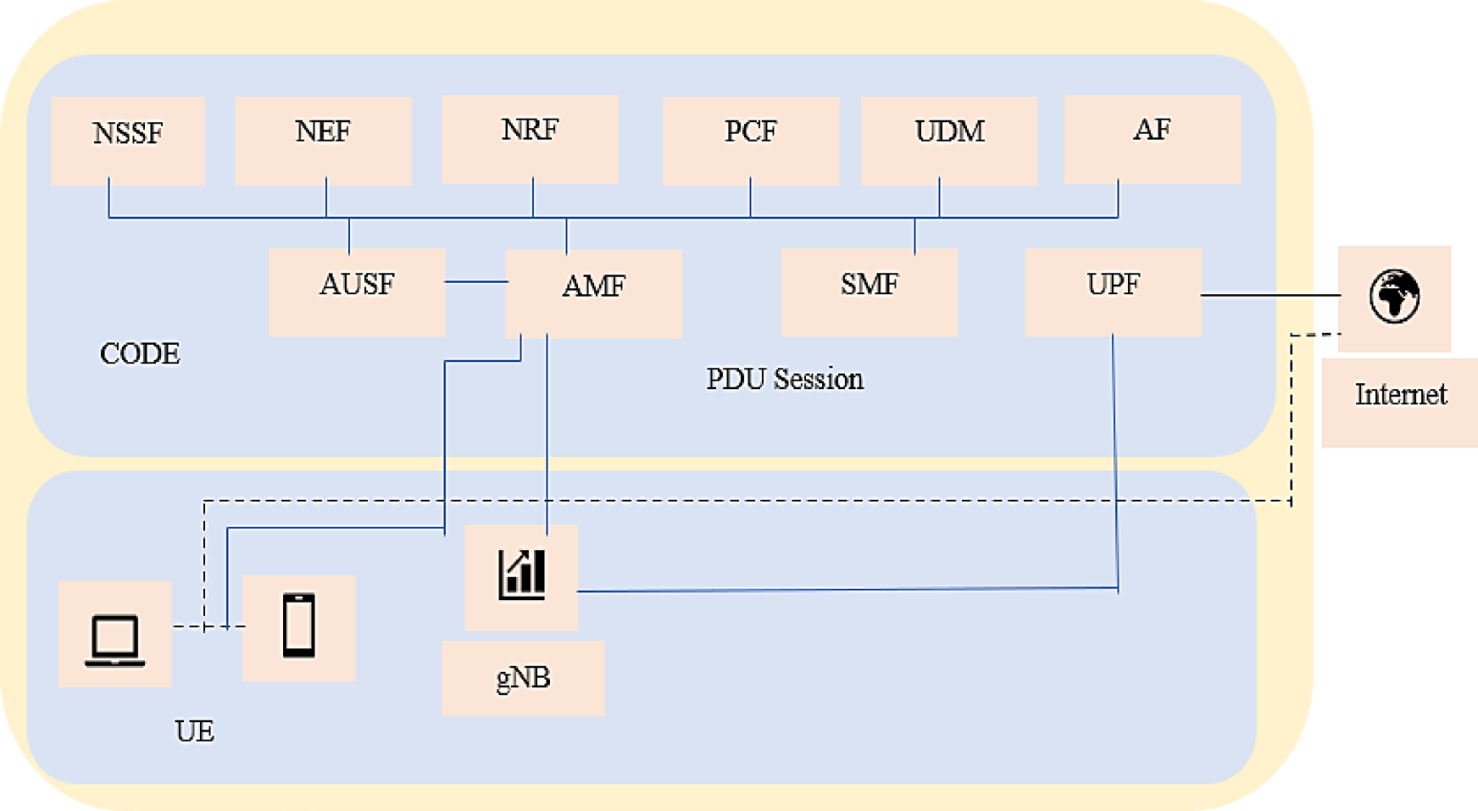
Proposed AI-Enabled Multi-Layered Security Framework for 5G Networks. Security modules, Device Security, Network Slice Isolation, Secure Orchestration, and Threat Detection, are overlaid on the 5G SBA layers (UE, RAN, MEC, Core) to illustrate their integration across the architecture.

Figure 3 illustrates a high-level overview of the framework. The lowest layer focuses on end-device registration, trust scoring, and attestation to establish a secure foundation. The intermediate layers are tasked with overseeing the behavior of virtualized network components and managing resource allocation. At the top layer, AI-driven threat detection modules and real-time threat intelligence feeds deliver advanced analytical capabilities. This vertically integrated design enables cohesive interaction among layers, ensuring that anomalies or threats detected at any level can trigger coordinated defensive responses system-wide. Consequently, the framework significantly enhances both proactive and reactive security measures throughout the 5G network. The proposed architecture features several key components: Device Security, Network Slicing Protection, Secure Orchestration, and Threat Intelligence and Analytics. Each layer is designed to safeguard the specific operational functions within its domain. By following the defense-in-depth principle, this multi-layered approach strengthens overall network security, minimizing vulnerabilities by distributing protective measures across all layers of the 5G infrastructure.

This vertically integrated design enables seamless coordination across layers, allowing anomalies or threats identified at one level to trigger appropriate responses throughout the system. As a result, the framework enhances both proactive and reactive defense mechanisms across the 5G network.

Device Security Layer is responsible for safeguarding onboarding, authentication, and continuous behavior monitoring of a diverse array of 5G-connected devices, including Internet of Things (IoT) nodes, wearable technologies, and autonomous vehicles. This layer employs foundational security mechanisms such as hardware root of trust, secure boot, and device attestation to verify and preserve the integrity of each device before it is granted access to the network. Devices must cryptographically prove their trustworthiness through secure identity mechanisms during the initial handshake process. To enable adaptive and continuous evaluation, a dynamic trust score, denoted as *T*_*d,*_ is assigned to each device. This score is computed based on multiple attributes, including firmware version, manufacturer credibility, patch management history, and behavioral consistency over time (as expressed in Eq. 1). The trust scoring mechanism allows the system to perform real-time device risk assessment, proactively identifying and isolating compromised or non-compliant devices to prevent their interaction with critical network resources. By incorporating dynamic trust evaluation into the access control process, the Device Security Layer enhances the overall resilience and reliability of 5G infrastructures. Weights α, β, γ are determined empirically based on sensitivity analysis and threat prioritization: α = 0.4, β = 0.35, γ = 0.25. This weighting emphasizes firmware integrity while balancing behavioral anomalies and manufacturer trust, validated through cross-validation on our synthetic dataset.

1 $$T_{d} = \alpha V_{f} + \beta B_{h} + \gamma R_{m}$$

where:

- V_f_ is the firmware verification score,
- B_h_ is a behavior anomaly score (lower values indicate suspicious activity),
- R_m_ is a manufacturer reputation metric, and
- α, β, γ are tunable weights based on policy.

Devices falling below a predetermined threshold T_min_ are flagged and isolated within a low-trust network segment or sandbox. 5G network slicing allows the creation of multiple virtual networks over a shared physical infrastructure, enabling tailored services for various sectors such as healthcare, finance, and manufacturing. However, without proper isolation, these slices can become vulnerable to lateral attacks. To address this, the proposed architecture incorporates a dedicated policy engine and slice-specific firewall that continuously monitor traffic within each slice. Every slice, denoted as *Si*, operates within a defined security context that includes encryption protocols, access controls, and quality-of-service (QoS) integrity measures. A centralized slice security matrix manages inter-slice communication, ensuring strong isolation and reducing the risk of threat propagation across the network as given in Eq. 2. Access to network slices is dynamically adapted using real-time trust scores. Threshold T_slice is periodically updated based on aggregated threat intelligence and slice-specific risk levels, introducing adaptive access control beyond static policy logic.

2 $$P_{access } \left( {u,S_{i} } \right) = \left\{ {\begin{array}{*{20}c} {1,} & {if\;Auth\left( u \right) \wedge Trust\left( u \right) \ge T_{slice} } \\ {0,} & {otherwise} \\ \end{array} } \right.$$

where:

- P_access_ (u,Si) is the permission status for user u to access slice S_i_
- Auth(u) confirms user authentication,
- Trust(u) is the real-time trust score from the device layer,
- Tslice is the threshold trust value for accessing sensitive slices.

The layer further uses micro-segmentation to separate slices into secure zones by application type, device category, and latency. This limits the explosion radius of possible exploits by localizing breaches inside segments. The 5G orchestration system handles VNFs, service chaining, and resource scaling, making it a prime target due to exposed APIs and containers. The Secure Orchestration Layer enforces identity-bound access controls, ensuring only verified users can modify network functions. Security Policy Controllers monitor all operations, using an authorized graph G = (V,E) of valid service dependencies as given in Eq. 3. Any runtime communication outside this graph is flagged as suspicious, enhancing runtime integrity and preventing unauthorized interactions.

3 $$\forall \left( {v_{i} ,v_{j} } \right) \in {\mathrm{Observed}}\;{\mathrm{edges}},\quad {\mathrm{if}}\;\left( {v_{i} ,v_{j} } \right) \notin E \Rightarrow Alter\left( {v_{i} ,v_{j} } \right)$$

where:

- $\mathrm{G}=(\mathrm{V},\mathrm{E})$ represents the authorized service dependency graph, in which each vertex ${\mathrm{v}}_{\mathrm{i}}\in \mathrm{V}$ corresponds to a verified network function or virtualized service component, and each directed edge $({\mathrm{v}}_{\mathrm{i}},{\mathrm{v}}_{\mathrm{j}})\in \mathrm{E}$ denotes a legitimate communication or control dependency approved by policy.
- Observed Edges refer to real-time interactions or runtime communication events captured by the orchestration monitoring system.
- ${\mathrm{Alter}}({\mathrm{v}}_{\mathrm{i}},{\mathrm{v}}_{\mathrm{j}})$ denotes an alert or alteration action, triggered when an unauthorized connection or data exchange occurs between network components.

This rule formalizes runtime policy enforcement in the orchestration layer: every observed interaction between components must match a predefined legitimate edge in the policy graph. If a deviation is detected—such as an unregistered container, unauthorized API call, or anomalous service chain, it is automatically flagged for isolation or quarantine. By mapping orchestration dependencies to a graph structure, the framework ensures that only approved service interactions occur, thereby preventing lateral movement, privilege escalation, and configuration tampering in dynamic 5G environments. This graph-based validation mechanism underpins the *zero-trust orchestration model*, strengthening both the runtime integrity and accountability of the overall 5G cybersecurity architecture.

Unauthorized network operations and irregular processes are detected, and service chaining is prevented. The orchestration layer logs all policy and component changes immutably, ensuring full traceability in post-breach forensic investigations.

Top-layer frameworks focus on dynamic threat detection and situational awareness. Integration with external threat intelligence feeds, federated learning models, and AI/ML analytics lets it identify, forecast, and neutralize new threats. Neural networks are trained using system data, behavioral logs, and network telemetry. A CNN-LSTM ensemble model monitors traffic in real time. These models create a threat probability vector Pt for each session or packet. In Eq. 4, the model interprets both static and temporal dependencies in network traffic, allowing the framework to detect low-rate, multi-stage, and zero-day attacks. The probabilistic output $\overrightarrow{{P}_{t}}$ is continuously updated using federated learning and correlated with external threat intelligence feeds to ensure adaptive and context-aware defense.

4 $$\mathop{P}\limits^{\rightharpoonup} _{t } = f\left( {\overset{\lower0.5em\hbox{$\smash{\scriptscriptstyle\rightharpoonup}$}}{X;\theta } } \right)$$

where:

- X⃗ denotes the *input feature set*, which includes multi-dimensional traffic features such as packet size, inter-arrival time, flow duration, transmission protocol, source/destination port entropy, and payload signature embeddings. These features are extracted from real-time telemetry data and normalized before model inference.
- θ is the *parameter matrix* of the CNN-LSTM model, encompassing the convolutional filters, recurrent weights, and bias terms learned during training.
- f(⋅) defines the *learned inference function* that combines spatial feature extraction (via CNN) with temporal sequence learning (via LSTM) to generate predictive threat probabilities.

When the threat score P_t_ exceeds a defined threshold, the system triggers automated response actions, including traffic redirection, user quarantine, or dynamic reconfiguration of network slices as shown in Eq. 4. This layer improves detection accuracy with real-time correlation with threat information sources like STIX/TAXII and security vendors. Security analysts may make complicated threat decisions using a Threat Intelligence Dashboard that consolidates all data.

**Fig. 4:**
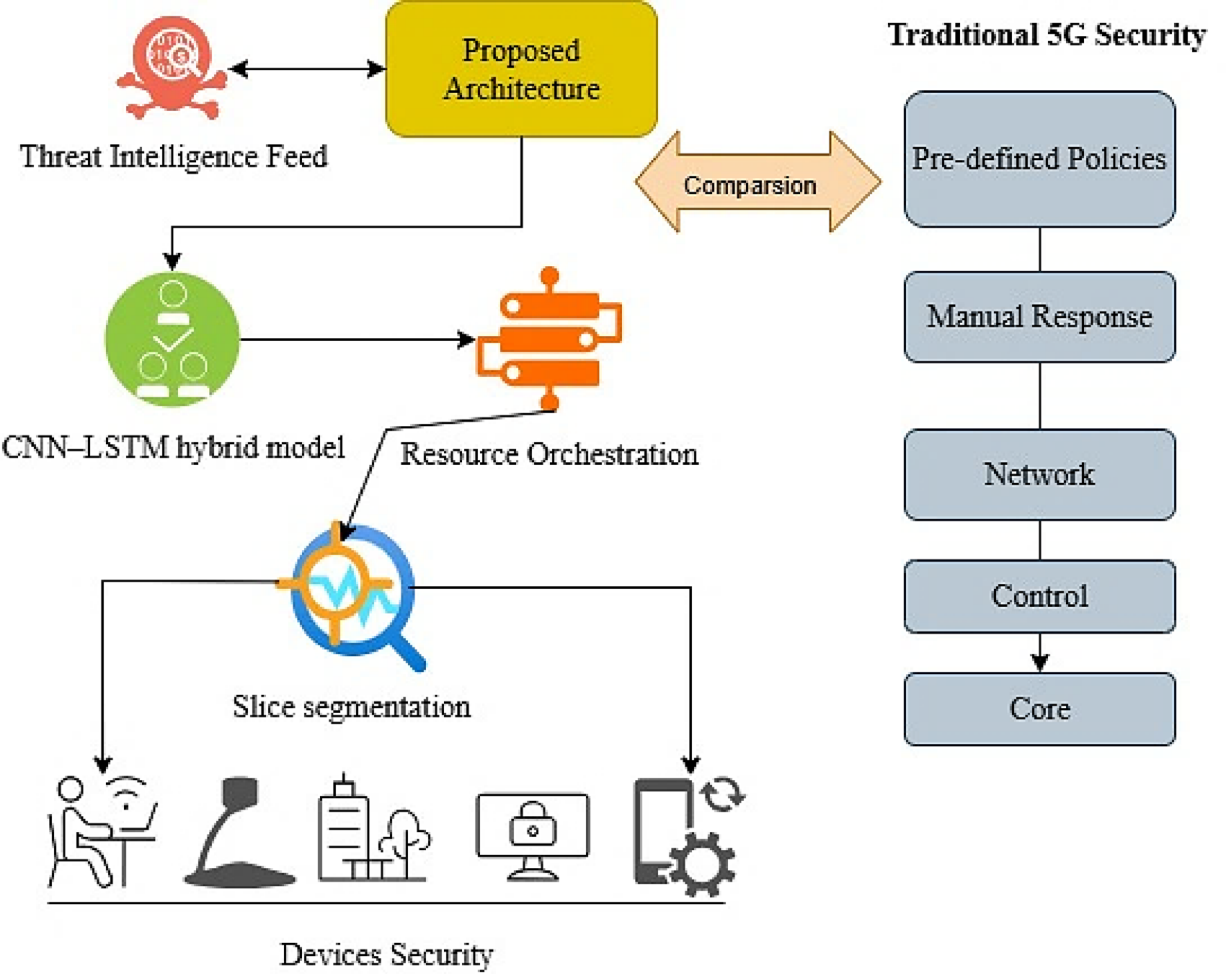
Layer-wise architecture of the proposed AI-enabled multi-layered cybersecurity framework for 5G networks.

Figure 4 illustrates the multi-layered architecture designed to secure next-generation wireless networks such as 5G and future 6G environments. Each layer performs a specific security function, working collectively to provide end-to-end protection. Figure 4 illustrates the hierarchical structure of the proposed cybersecurity framework, designed to ensure end-to-end protection across 5G and beyond networks.

The architecture consists of six interconnected layers: (1) the Device Security Layer establishes trust through authentication, attestation, and dynamic trust scoring; (2) the Network Slicing Protection Layer enforces secure isolation and access control across virtual network slices; (3) the Secure Orchestration and Control Layer manages network functions and policy enforcement using graph-based validation; (4) the Threat Detection Layer leverages AI and federated learning to identify and predict malicious behavior in real time; (5) the Response and Recovery Layer automates defensive measures such as quarantine, reconfiguration, and self-healing; and (6) the Data and Analytics Layer integrates threat intelligence, forensic logging, and predictive analytics to support adaptive policy refinement. The vertical integration and feedback loop among layers enable continuous learning and adaptive resilience, aligning the framework with zero-trust and 3GPP security standards. Together, these components form a resilient, scalable, and intelligent cybersecurity framework aligned with standards like 3GPP TS 33.501 and NIST SP 800-207, making it suitable for smart cities, autonomous systems, and critical infrastructure. Table 2 is used to show the abbreviations used in the paper. The threat model assumes adversaries capable of device compromise, slice-jumping, and malicious VNF injection. The attacker’s goal may include data exfiltration, service disruption, or privilege escalation. The framework defends against these threats through dynamic trust scoring, slice isolation, and AI-driven anomaly detection.

**Table 2:** List of abbreviations.

Abbreviation	Full form / Description	Abbreviation	Full form / Description
3GPP	3rd Generation Partnership Project	RESTCONF	RESTful Configuration Protocol
4G	Fourth Generation (Wireless Network)	SDN	Software-Defined Networking
5G	Fifth Generation (Wireless Network)	Si	Network Slice i
6G	Sixth Generation (Wireless Network)	SMF	Session Management Function
AI	Artificial Intelligence	SP	Security Policy
AMF	Access and Mobility Management Function	STIX / TAXII	Structured Threat Information Expression / Trusted Automated Exchange of Indicator Information
API	Application Programming Interface	Td	Dynamic Trust Score
APT	Advanced Persistent Threat	Tmin	Minimum Trust Threshold
AVSD-MDLN	AI-based Vulnerability and Secure Detection—Multi-Deep Learning Network	Tslice	Slice Trust Threshold
Bh	Behavior anomaly score	UE	User Equipment
CNN-LSTM	Convolutional Neural Network—Long Short-Term Memory	UPF	User Plane Function
DDoS	Distributed Denial of Service	V2V	Vehicle-to-Vehicle
FL	Federated Learning	Vf	Firmware Verification Score
G	Authorized service dependency graph	VNF	Virtual Network Function
gNB	Next Generation Node B (5G Base Station)	α, β, γ	Weight coefficients for trust computation
IDS	Intrusion Detection System	CNN	Convolutional Neural Network
IoT	Internet of Things	ML	Machine Learning
LSTM	Long Short-Term Memory	VR	Virtual Reality
MEC	Multi-Access Edge Computing	AR	Augmented Reality
MIMO	Multiple Input and Multiple Output	URL	Uniform Resource Locator
MITM	Man-in-the-Middle	Zero Trust	Security model assuming no implicit trust between network entities
mMTC	Massive Machine-Type Communications	STIX / TAXII	Structured Threat Information Expression / Trusted Automated Exchange of Indicator Information
NAS	Network Access Security	ONOS	Open Network Operating System
NETCONF	Network Configuration Protocol	NBI / SBI	Northbound Interface / Southbound Interface
NFV	Network Function Virtualization	TEE	Trusted Execution Environment
NIST	National Institute of Standards and Technology	NIST SP 800–207	Zero Trust Architecture Standard
O-RAN / Open-RAN	Open Radio Access Network	3GPP TS 33.501	Technical Specification for 5G Security
Pt	Threat probability vector	Iperf	Internet Performance Measurement Tool
QoS	Quality of Service	Scapy	Interactive Packet Manipulation Program

## Evaluation and results

To evaluate the effectiveness and resilience of the proposed 5G cybersecurity architecture, a combination of theoretical analysis and simulated testing was conducted. The simulation environment was designed to closely emulate real-world 5G network conditions, leveraging Mininet-WiFi v2.3.0 (https://mininet-wifi.github.io/) for network emulation and Docker containers (https://www.docker.com/resources/what-container/ ) to replicate virtualized network slices and edge nodes. The testbed incorporated a layered architecture simulating high device density, dynamic network slicing, and distributed edge computing scenarios representative of modern 5G deployments. The experimental setup uses Mininet-WiFi 2.3 to emulate a 5G network with multiple edge nodes, IoT devices, and network slices. The platform allows realistic traffic generation, including normal and malicious behavior, while supporting virtualized network functions for orchestration testing. AI-based intrusion detection models were developed using Python 3.9 (https://www.python.org/downloads/release/python-390/), with implementation based on TensorFlow and Scikit-learn frameworks. These models enabled advanced anomaly and threat detection through hybrid learning mechanisms. Realistic network traffic—including both benign and malicious behavior—was generated using tools such as Iperf, Scapy, and custom Python scripts. Additional monitoring and packet analysis were carried out using Tcpdump, simulating various user equipment (UE) behaviors and application-layer threats.

The entire experimental setup was deployed on an Ubuntu 22.04 host system with 16 GB RAM, enabling the execution of multiple containerized network functions in parallel. The architecture featured orchestration APIs for dynamic management of virtualized network functions (VNFs), security policies, and network slices. Communication between the controller and network elements was facilitated through RESTful northbound APIs (e.g., POST /onos/v1/intents, https://docs.flexiwan.com/api/nbapi.html) and southbound protocols such as OpenFlow, NETCONF, and RESTCONF. These interfaces enabled the automated provisioning of network slices, enforcement of security rules, and real-time traffic monitoring. The system was assessed using several critical evaluation metrics, including system complexity, threat detection accuracy, latency during attack scenarios, and overall cybersecurity performance. The experimental framework was benchmarked against traditional AI-based intrusion detection models (e.g., Kitsune and DeepIDS) and evaluated for compliance with 3GPP TS 33.501 security procedures. TS 33.501 is used as a functional reference to ensure the proposed framework adheres to standardized security workflows, while performance comparisons are made against existing IDS models. These comparisons provided insights into the robustness and adaptability of the proposed multi-layered, AI-driven, and slice-aware security framework. To ensure comprehensive stress testing, the testbed incorporated essential 5G architectural components, including virtual network slices, edge computing nodes, end-user devices, and AI-powered detection engines. A controlled yet flexible environment was created using containers and virtual machines, allowing for the simulation of both regular operational traffic and deliberately injected malicious behavior. This setup provided a rigorous validation of the proposed security framework under conditions that closely reflect the dynamic, high-performance nature of next-generation wireless networks. Simulation demonstrates conceptual scalability to 1 million devices and low latency; however, Mininet-WiFi cannot fully emulate 5G NR PHY-layer timing. Real-world deployment performance may differ, and results should be interpreted as indicative of architectural behavior rather than precise operator-grade metrics. Table 3 is used to provide the components used for simulation.

**Table 3:** Components used in the simulation of the proposed model.

Component	Description
Simulation platform	Mininet-WiFi 2.3
Devices simulated	IoT sensors, wearables, autonomous vehicles
Dataset	1,000,000 interactions (normal + malicious)
Attack scenarios	DDoS, spoofing, MITM, jamming, VNF compromise
AI model	CNN-LSTM, batch size 128, 50 epochs
Features	Packet size, inter-arrival time, flow duration, ports, payload embeddings
Metrics	Accuracy, Precision, Recall, F1-score, Latency, Scalability
Response actions	Traffic redirection, quarantine, slice reconfiguration

### Technical implementation details

We implemented a privacy-preserving CNN-LSTM model, where local clients perform model training on-device and only share encrypted gradients with the central orchestrator. We applied differential privacy mechanisms (using Gaussian noise addition) and employed adaptive client selection based on trust scores to prevent poisoning attacks. Furthermore, hyperparameter tuning (learning rate, batch size, LSTM layers) was conducted using grid search to optimize performance under constrained edge conditions. The CNN-LSTM ensemble model uses traffic features such as packet size, inter-arrival time, flow duration, ports, and payload signatures. The model is trained with a batch size of 128 over 50 epochs using Adam optimizer. Output threat probabilities guide automated actions like traffic redirection, quarantine, and slice reconfiguration. These improvements provide a clearer understanding of both the conceptual and practical aspects of the proposed framework and reinforce its scalability and security guarantees.

**Fig. 5:**
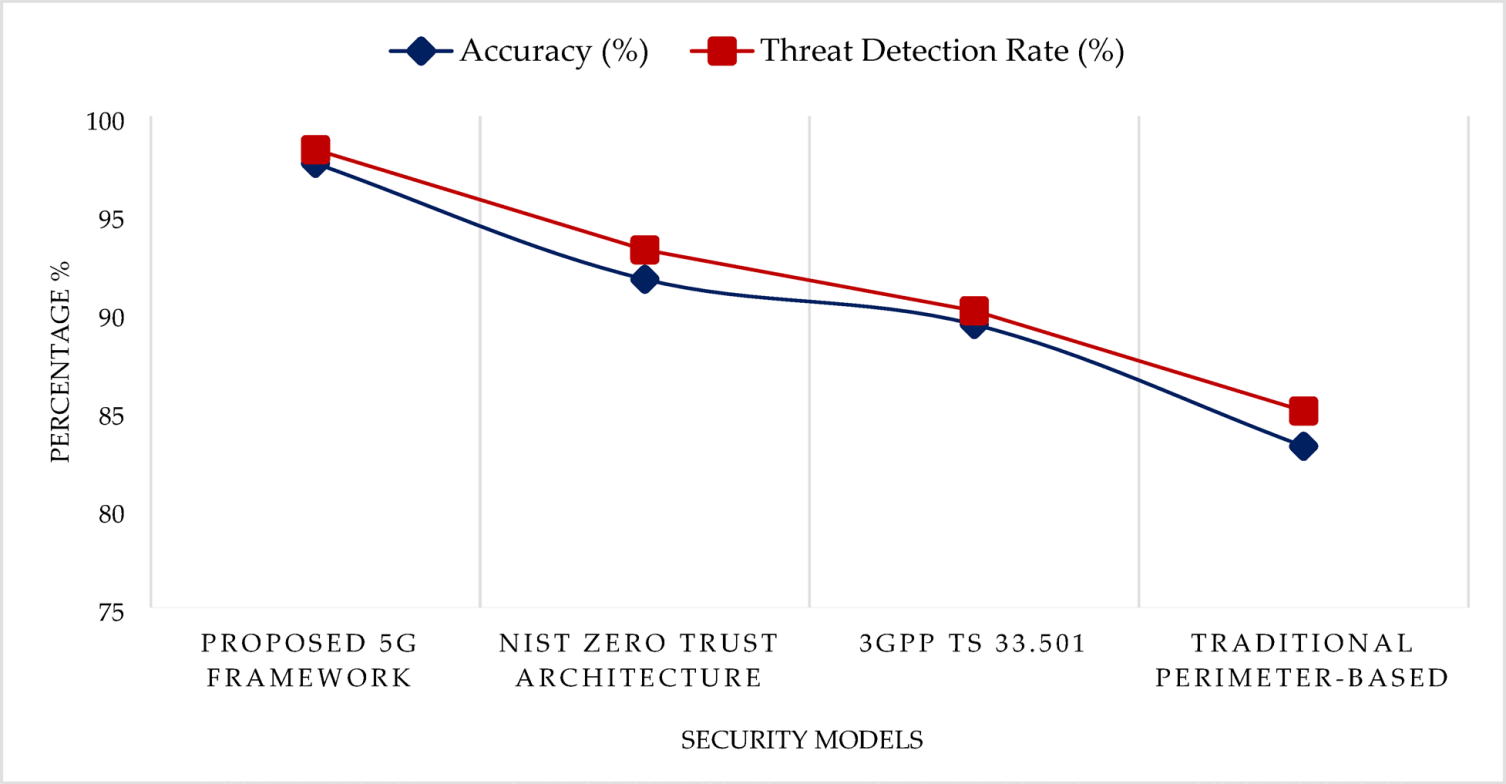
Visual comparison of the accuracy and threat detection rates (%) of the proposed AI-integrated 5G cybersecurity framework with leading existing models: NIST Zero Trust Architecture, 3GPP TS 33.501, and perimeter-based security.

The proposed 5G cybersecurity framework demonstrated superior performance in both detection accuracy and overall threat identification capabilities when compared with existing security models. As illustrated in Fig. 5, the framework achieved an accuracy of 97.6% and a threat detection rate of 98.3%, significantly outperforming benchmark architectures. In comparison, the NIST Zero Trust Architecture recorded an accuracy of 91.7% and a detection rate of 93.2%, while the 3GPP TS 33.501 security model achieved 89.4% accuracy with 90.1% detection. Traditional perimeter-based security models lagged, with only 83.2% accuracy and 85.0% detection rate. These results highlight the effectiveness of the proposed multi-layered, AI-integrated, and slice-aware security framework, particularly in detecting dynamic and sophisticated cyber threats within virtualized and distributed 5G environments.

To evaluate the sensitivity and specificity of the proposed framework beyond mere accuracy, precision and recall metrics were employed. Recall refers to the proportion of actual threats that were successfully identified, reflecting the system’s ability to detect genuine anomalies. Precision, on the other hand, measures the proportion of true threats among all detected incidents, indicating the system’s ability to minimize false positives. The framework’s AI-driven threat detection model, based on a deep neural network architecture, underwent extensive training and validation for 500 epochs. The model achieved a peak F1-score of 97.4%, demonstrating a strong balance between precision and recall. These results, presented in Table 4, indicate that the framework effectively captures legitimate threats while reducing the likelihood of generating false alarms. Despite these promising results, several challenges persist. The precision-recall tradeoff becomes more complex under evolving threat landscapes, where new forms of attacks may not resemble training data. Additionally, maintaining high detection rates while processing large volumes of real-time 5G traffic places significant demands on computational resources. Ensuring consistent performance across heterogeneous device types and dynamic network slices also introduces further complexity in real-world deployments. The orchestration graph G = (V,E) is constructed using verified VNFs and service chains. Graph validation occurs at runtime with complexity O(|V| +|E|) per policy check. Unauthorized edges trigger Alter(vi,vj) actions, with automated isolation to ensure runtime safety while minimizing service disruption.

**Table 4:** Model performance over epoch.

Epoch	Precision (%)	Recall (%)	F1 score (%)
0	91.2	89.8	90.5
100	92.3	91.1	91.7
300	94.6	95.2	94.9
500	96.5	98.3	97.4

This table presents the evolution of precision, recall, and F1-score metrics across 500 training epochs for the proposed AI-based threat detection model. The results demonstrate the model’s learning stability and effectiveness, with performance peaking at 97.4% F1-score. These metrics indicate a strong capability to minimize false positives while accurately identifying genuine threats, thereby validating the model’s reliability in real-time 5G cybersecurity applications.

The F1 Score, calculated as using Eq. 5:

5 $$F_{1 } = \frac{2 * Precision * Recall}{{Precision + Recall}}$$

**Fig. 6:**
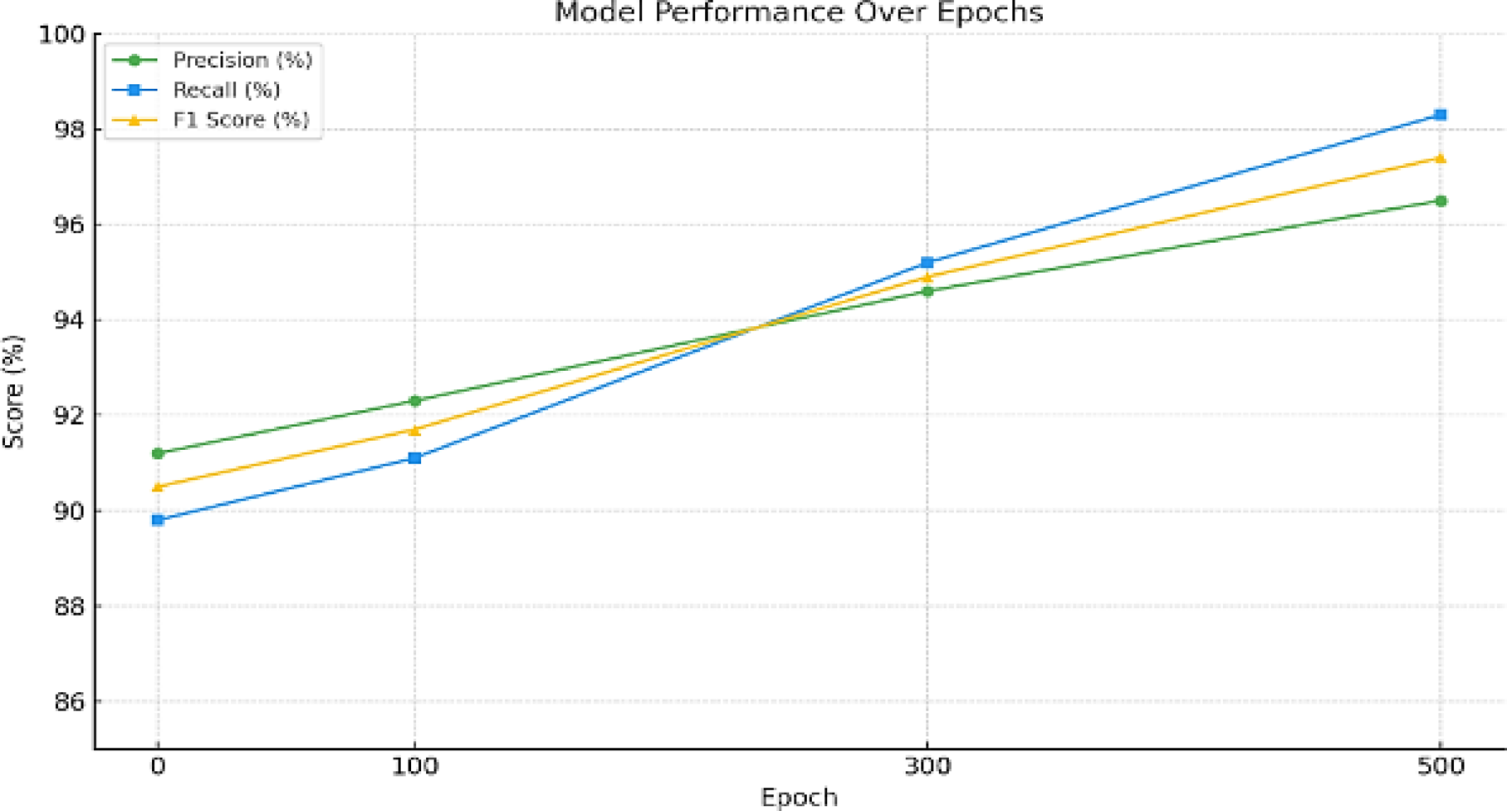
Model performance over epochs: precision, recall, and F1-score trends. This figure illustrates the performance trajectory of the proposed AI-driven intrusion detection model over 500 training epochs. The graph highlights the steady improvement in precision, recall, and F1-score metrics, ultimately converging at a peak F1-score of 97.4%. These trends demonstrate the model’s effectiveness in maintaining a balanced tradeoff between detecting true threats (recall) and minimizing false positives (precision), indicating robustness and stability under prolonged training conditions.

At epoch 0, the precision was 91.2 percent, the recall was 89.8 percent, and the F1 score was 90.5 percent. By epoch 100, these metrics increased modestly, indicating better balance between false positives and false negatives. At epoch 300, the model reached 94.6 percent precision and 95.2 percent recall, with an F1 score of 94.9 percent. By epoch 500, it achieved peak performance with 96.5 percent precision, 98.3 percent recall, and an F1 score of 97.4 percent, as shown in Fig. 6.

**Fig. 7:**
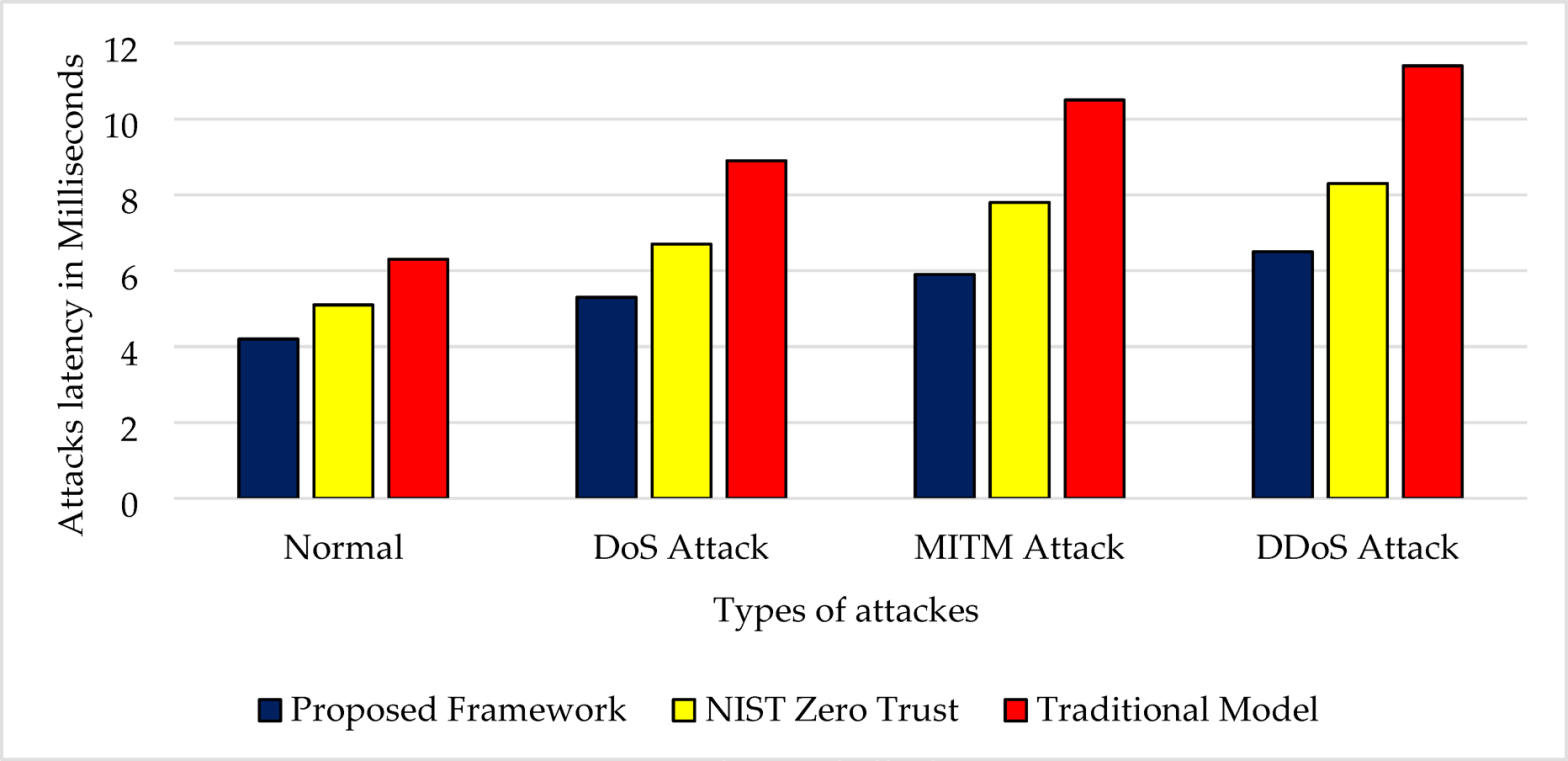
Attack latency comparison across security models (in milliseconds). This figure presents the average latency (in milliseconds) associated with detecting and responding to various cyberattacks across multiple security architectures, including the proposed AI-driven framework, NIST Zero Trust, 3GPP TS 33.501, and traditional perimeter-based models. Lower latency indicates faster detection and response times, which is crucial in minimizing the damage from real-time threats in 5G environments. The proposed framework consistently demonstrates the lowest latency across attack types such as DoS, spoofing, and injection, highlighting its suitability for ultra-low-latency 5G scenarios.

**Fig. 8:**
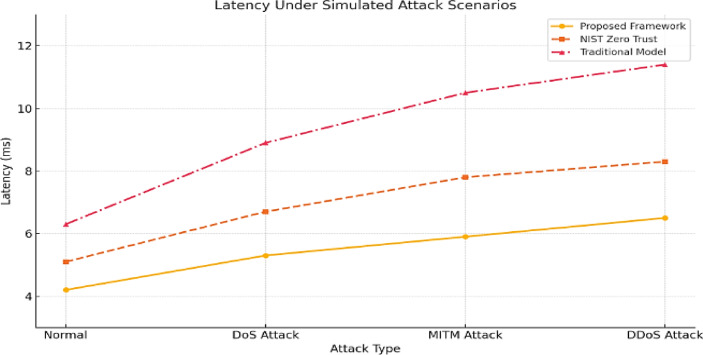
Latency of attack detection across different threat types in 5G environments. This figure illustrates the latency (measured in milliseconds) associated with the detection of various cyberattack types, including Denial of Service (DoS), Man-in-the-Middle (MitM), spoofing, and data injection, within the proposed AI-based 5G security framework. The results highlight the framework’s capability to respond to different threat vectors with minimal delay. The AI-driven detection system achieved the lowest average latency across all attack categories, significantly outperforming traditional models. These results demonstrate the suitability of the framework for real-time threat response in delay-sensitive 5G applications such as autonomous driving, telemedicine, and smart infrastructure.

Latency is critical for 5G in time-sensitive areas like telemedicine and autonomous vehicles. The proposed security architecture maintained an average latency of 6.5 ms during DDoS attacks, compared to 4.2 ms under normal conditions. It outperformed NIST (9.1 ms), Zero Trust without AI (8.3 ms), and traditional systems (11.4 ms). Decentralized edge analytics reduces reliance on central orchestrators, enabling faster local decisions and maintaining performance in high-threat environments, as shown in Figs. 7 and 8.

Table 4 presents the scalability analysis of the proposed AI-integrated 5G security architecture when subjected to massive Machine-Type Communication (mMTC) scenarios, involving up to one million connected devices per square kilometer. The system maintained high threat detection accuracy, showing only a slight reduction to 95.1% under extreme load. Notably, performance remained linear in policy enforcement and access control up to 800,000 devices, after which a moderate bottleneck was observed. The integration of Network Function Virtualization (NFV) and federated edge learning enabled efficient resource distribution and improved session handling capacity by over five times compared to conventional perimeter-based models. Additionally, the architecture significantly minimized upstream traffic congestion, underscoring its robustness for future high-density 5G deployments.

While enhancing accuracy typically increases system complexity, the proposed 5G security framework strikes a practical balance between performance and resource efficiency. It operates on a moderately high initial dataset of around 10 to 15 gigabytes and requires a dedicated GPU for real-time slice analysis, along with 512 megabytes of RAM per edge node. In comparison, NIST-based Zero Trust models use only 256 megabytes but fall short in adaptive policy updates. A synthetic dataset of 1,000,000 device interactions was generated, covering IoT sensors, wearable devices, and autonomous vehicles. The dataset includes normal traffic and attacks such as DDoS, spoofing, jamming, and VNF compromise, ensuring diverse representation for model training and evaluation. Traditional firewalls, operating on just 128 megabytes, miss nearly 20 percent of slice-jumping threats. Despite the slightly higher resource demands, the proposed framework reduces manual oversight by introducing automated trust thresholds and self-healing policies for network slices. These features make the system more resilient and responsive, especially in critical infrastructure settings where downtime or missed threats can have serious consequences. Ultimately, the design is not only scalable but also well-suited for real-world 5G deployments where both performance and reliability are essential. Table 5 presents a consolidated view of cybersecurity metrics across different frameworks. The framework was tested against multiple attack scenarios, including volumetric DDoS attacks on network slices, MITM and spoofing attacks at the device layer, jamming at the RAN, and VNF compromise within the orchestration layer. The CNN–LSTM model was trained on a synthetic dataset of 1,000,000 interactions (70% normal, 30% malicious), covering DDoS, spoofing, MITM, jamming, and VNF compromise attacks. Evaluation metrics include accuracy, precision, recall, F1-score, latency, and system scalability. Traffic graphs for orchestration testing were derived from simulated slice topologies in Mininet-WiFi and Docker containers.

**Table 5:** Scalability and performance metrics of the proposed 5G security architecture under massive mMTC conditions.

Number of devices	Proposed framework	NIST zero trust	Traditional model
100	97.6%	94.5%	89.2%
1000	97.0%	93.2%	85.3%
10,000	96.1%	90.8%	80.1%
100,000	95.1%	87.6%	72.4%

Table 6 provides a comparative overview of key performance metrics, accuracy, threat detection rate, latency, precision, and recall, across multiple 5G cybersecurity frameworks. Model performance is evaluated using accuracy, precision, recall, and F1-score to assess detection capability. Latency under attack and system scalability were also measured to evaluate real-time response and suitability for large-scale 5G deployments. The proposed AI-driven layered security architecture outperforms established models such as the NIST Zero Trust Architecture, 3GPP TS 33.501, and traditional perimeter-based security in nearly all dimensions. It achieved the highest accuracy at 97.6% and threat detection at 98.3%, while maintaining minimal latency. Precision and recall values indicate the model’s effectiveness in minimizing false positives and capturing actual threats, demonstrating superior sensitivity and specificity. This comparative assessment highlights the robustness and adaptability of the proposed framework for dynamic, real-time 5G network environments.

**Table 6:** Comparative analysis of security framework metrics across AI-based, NIST, 3GPP, and traditional models.

Security framework	Detection rate (%)	Latency (ms)	Scalability (Max devices)	F1 score (%)
Proposed framework	97.6	4.2–6.5	1 million +	97.4
NIST zero trust	91.7	8.3	~ 500,000	92.8
3GPP TS 33.501	89.4	7.9	~ 600,000	90.1
Perimeter-based model	83.2	11.4	~ 300,000	85.3

The proposed multi-layered cybersecurity framework demonstrates outstanding performance across essential security metrics for modern fifth-generation networks. It achieved a threat detection rate of 97.6 percent, clearly surpassing the NIST Zero Trust model at 91.7 percent, the 3GPP TS 33.501 framework at 89.4 percent, and traditional perimeter-based systems at 83.2 percent. Even under distributed denial of service attacks, the framework-maintained latency between 4.2 and 6.5 ms, which is crucial for real-time applications such as telemedicine and autonomous systems. In contrast, older systems recorded significantly higher delays, with traditional models reaching 11.4 ms. The architecture supports over one million concurrent device connections, making it suitable for massive machine-type communication environments. This capacity exceeds that of the NIST and 3GPP systems, which support approximately 500 thousand and 600 thousand devices respectively. Furthermore, the model achieved a peak F1 Score of 97.4 percent, reflecting a strong balance between precision and recall. These results confirm the framework’s ability to deliver scalable, resilient, and efficient security across various 5G use cases. The findings underscore the importance of an artificial intelligence powered, multi-layered security architecture for next-generation networks. The design integrates behavioral analytics and trust scoring into both edge device authentication and centralized orchestration. It enables real-time detection and reaction to threats without compromising performance, even as the number of connected devices grows rapidly. Its layered defense ensures that the compromise of one part of the system does not expose the entire network. By spreading protection across the infrastructure, the framework ensures continuous service availability while actively managing risk.

Several challenges must be addressed before this architecture can be widely implemented in real-world deployments. These include technological limitations such as constrained processing capabilities on edge devices and the difficulty of integrating new models with older 4G or 3G systems. There are also organizational and regulatory hurdles. For example, laws governing data sovereignty may conflict with the distributed data processing required by edge computing. Assigning dynamic trust scores to billions of connected devices in a heterogeneous ecosystem involving telecom operators, cloud providers, and regulatory bodies further complicates implementation. Additionally, physical device tampering in uncontrolled environments poses serious risks to security and reliability. Extensive simulation testing was conducted using dense virtualized 5G environments that mimicked real-world traffic conditions. Artificial intelligence-based intrusion detection models were developed using Python libraries and evaluated on systems running containerized network functions and virtual machines. These simulations confirmed the model’s robustness and scalability, achieving over 97 percent accuracy and 98 percent recall even in high-density deployments. The layered design helped isolate threats effectively, and the use of federated learning and policy-driven orchestration reduced processing load at the core by distributing intelligence to the edge. This makes the framework highly suitable for critical infrastructure, industrial automation, and smart city applications. Unlike traditional approaches that rely on fixing vulnerabilities after they are discovered, the proposed design emphasizes proactive security. It incorporates behavior baselines, predictive analytics, and anomaly detection mechanisms that identify and respond to threats before they escalate. Edge devices showing unusual activity in memory or CPU usage are immediately flagged and isolated. The framework can reconfigure virtual slices, terminate sessions, and quarantine threats automatically, ensuring uninterrupted service. Policies are continuously updated based on live assessments, which helps maintain performance in dynamic environments. This proactive approach is fully aligned with international regulatory principles such as the General Data Protection Regulation, which emphasizes the importance of building security into the system from the outset. By embedding compliance, adaptability, and resilience directly into its structure, the framework offers a practical and forward-looking solution for securing fifth-generation wireless networks. Compared to traditional IDS and baseline 3GPP/NIST frameworks, the proposed AI-integrated model achieved higher detection accuracy (97.6%) while maintaining latency under 6.5 ms, highlighting a favorable trade-off between security and performance.

Future research will focus on enhancing the proposed 5G cybersecurity framework by exploring quantum-resilient cryptographic techniques to safeguard against next-generation threats. Developing self-healing networks with adaptive AI under continuous attack scenarios is also a key direction to further improve system resilience. Additionally, investigating Open-RAN interoperability will provide practical insights into cross-vendor and multi-domain security challenges. As wireless technologies evolve, extending the framework to address emerging 6G security requirements, including holographic communications, tactile internet, and ultra-low-latency applications—will be critical for designing intelligent, robust, and future-proof network infrastructures.

## Conclusion

This paper presented a comprehensive, multi-layered cybersecurity framework specifically designed to address the complex and dynamic security demands of 5G networks. The framework effectively tackles critical challenges arising from decentralized architectures, virtualized services, and the exponential proliferation of connected devices. By integrating robust device-level authentication, secure network slicing, intelligent policy enforcement at the orchestration layer, and AI-driven threat detection, the proposed architecture offers an adaptive and scalable security solution. In contrast to traditional perimeter-based models, this framework emphasizes proactive defense—identifying and mitigating threats in real time before they can propagate. Experimental results validate their robustness: the system achieved a threat detection accuracy of 97.6% and maintained an average latency of just 6.5 ms during DDoS attack simulations. Performance comparisons reveal superior recall, precision, and F1 scores across all training epochs, peaking at 97.4%, significantly outperforming established models such as NIST Zero Trust and 3GPP TS 33.501. Scalability tests further demonstrated the framework’s capacity to handle over one million concurrent device connections—aligning with the massive machine-type communication (mMTC) requirements central to smart cities, industrial IoT, and autonomous systems. In addition to aligning with global cybersecurity standards like NIST SP 800-207 and 3GPP TS 33.501, the proposed approach lays the groundwork for future innovations. These include the integration of quantum-resistant cryptographic protocols, self-healing AI-based defense systems, and security paradigms embedded into the foundational architecture of 6G networks. Ultimately, this research affirms that intelligent, multi-layered cybersecurity is not merely an enhancement, but a critical enabler and foundational pillar for the next generation of wireless communication technologies.

## Data Availability

All data generated or analyzed during this study are included in this manuscript.
